# A sequentially metastatic gastric and jejunal cancer originating from colon cancer: A case report

**DOI:** 10.1016/j.ijscr.2020.05.009

**Published:** 2020-05-19

**Authors:** Woo Yong Lee, Hye Kyeon Lee

**Affiliations:** a Department of Surgery, Seoul Paik Hospital, Inje University College of Medicine, 9, Mareunnae-ro, Jung-gu, Seoul 100-032, Republic of Korea; b Department of Pathology, Seoul Paik Hospital, Inje University College of Medicine, 9, Mareunnae-ro, Jung-gu, Seoul 100-032, Republic of Korea

**Keywords:** Primary colon cancer, Metastatic gastric and jejunal cancer, Blind loop of Roun-en-Y anastomosis

## Abstract

**Introduction:**

Metastatic intraluminal cancer arising from gastrointestinal tract cancer is very rare. In this report, we describe a case of an 82-year-old man with sequentially metastatic gastric and jejunal cancer originating from primary colon cancer.

**Presentation of case:**

An 82-year-old Korean male patient with a history of right colon cancer was initially treated with extended right hemicolectomy. The tumour was classified as pT3N0M0 and stage II. After nine months, a gastroscopy revealed an infiltrating ulcerative mass in the cardia of the stomach, a colonoscopy revealed no specific findings in the previous operation site, and a positron emission tomography-computed tomography scan revealed no distant metastasis. The patient underwent radical total gastrectomy, and the final pathologic diagnosis was T3N2M0, stage IIIA. During follow-up without chemotherapy, a gastroscopy revealed tumours in the blind jejunal loop of Roux-en-Y anastomosis, and an endoscopic biopsy confirmed adenocarcinoma. The patient then underwent segmental resection of the blind loop jejunal cancer. Finally, further pathological examination of the resected specimen confirmed that the lesion represented a sequentially metastatic gastric and jejunal cancer originating from colon cancer.

**Discussion:**

The exact mechanism of intraluminal metastasis of gastrointestinal tract cancer is not known. Immunohistochemical staining might prove useful in sequentially metastatic cases when a differential diagnosis must be assessed on consecutive biopsies.

**Conclusion:**

Although intraluminal metastasis of gastrointestinal tract cancer is very rare, researchers should be aware of this uncommon intraluminal metastasis.

## Highlights

• First case of metastatic intraluminal cancer from gastrointestinal tract cancer.
• Immunohistochemical staining may be useful in sequentially metastatic cancer cases.
• Adenocarcinomas were derived from colon cancer with CK7, CK20, and CDX2 staining.
• When an uncommon carcinoma develops researchers should consider previous ones.

## 1 Introduction

A 2018 worldwide study revealed colorectal cancer to be the second and third most common cancer in women and men, respectively [1]. As is the case in other carcinomas, the common cause of death in colorectal cancer is metastasis. Colorectal cancer usually metastasizes to the thoracic cavity, liver, peritoneum, or combinations of these sites. Gastrointestinal metastasis is most often superficial due to peritoneal carcinomatosis, and intraluminal metastasis of colorectal cancer is extremely rare. However, we present an extremely rare case of sequentially gastric and jejunal metastases from colon cancer without peritoneal carcinomatosis in an 82-year-old man in Korea. This paper has been reported in line with the SCARE criteria [2].

## 2 Case presentation

**Fig. 1:**
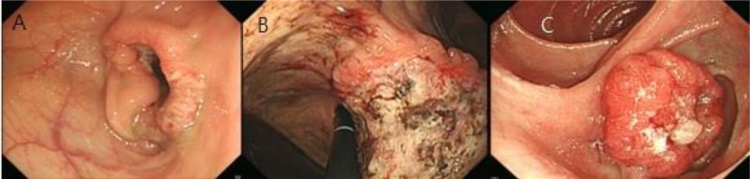
Endoscopy finding. A. Ulcerofungating mass (5*5 cm) in hepatic flexure (colon). B. Large infiltrating ulcerative mass (7*7 cm) in mid-to-high body in the lesser curvature side (stomach). C. Ulcerative mass (2.3*2.5 cm) in blind loop with Roux-en-Y anastomosis (jejunum).

**Fig. 2:**
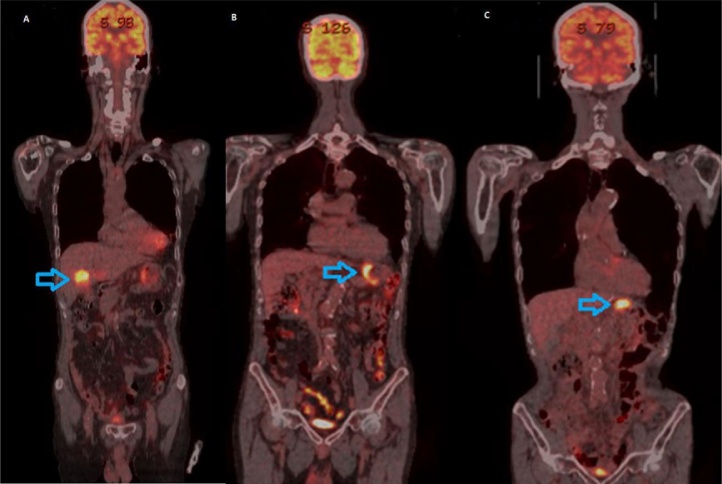
Positron emission tomography–computed tomography (PET-CT). A. Colon cancer (SUV; 12.6) without evidence of distant metastasis (cT3N0M0). B. Gastric cancer (SUV; 11.8) in upper body of stomach without evidence of distant metastasis (cT3N1M0). C. Local recurrence (SUV; 9.4) of previous stomach cancer without metastasis.

**Fig. 3:**
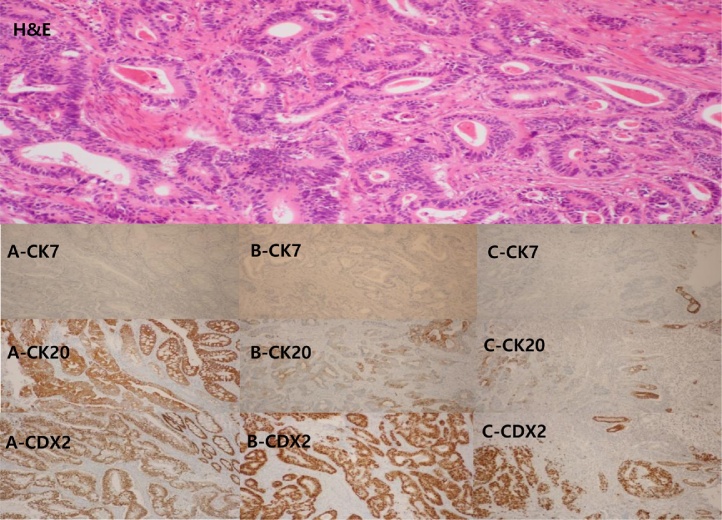
Histologic examination of biopsy. A: colon, B: stomach, C: jejunum. Colon cancers including metastatic gastric and jejunal cancers were all relatively moderate differentiated adenocarcinomas, intestinal type (Gastric ca, H&E, ×100). Homogenous, high levels (3+) of CDX2 expression (×100) were found in primary colonic and metastatic, as well as gastric and jejunal adenocarcinomas. The immunohistochemical staining for cytokeratin (CK) profile (×100) demonstrated the same immunoreactivities in both primary colonic and metastatic, as well as gastric and jejunal adenocarcinomas: CK 20 (+) & CK 7 (−).

This is the case of an 82-year-old male with a known history of gastric cancer. He had undergone right hemicolectomy for colon cancer nine months ago (Fig. 1A), and his final pathologic examination showed moderately differentiated adenocarcinoma that had invaded into pericolic tissues (T3). There was no metastasis to regional lymph nodes (N0), and the tumour was classified as pT3pN0 (0/18) M0, stage II. Further treatment including chemotherapy was recommended, but the patient declined adjuvant chemotherapy. During the follow-up period, there were no specific findings. At the nine-month postoperative follow-up study, a gastroscopy revealed a large infiltrating ulcerative mass (7*7 cm) on the high body of the stomach (Fig. 1B), but a colonoscopy revealed no specific findings at the previous operation site. A computed tomography scan of the abdomen showed a tumour in the lesser curvature of the gastric high body and enlarged perigastric lymph nodes. The CEA and CA19-9 levels were within the normal limit, and other laboratory examinations indicated no specific findings. A positron emission tomography-computed tomography (PET-CT) scan only showed gastric uptake (maximum standardized uptake value (SUV), gastric cardia-11.8) without evidence of any other metastasis (Fig. 2B). The patient underwent total gastrectomy with Roux-en-Y anastomosis and D2 lymph node dissection. Pathologic examination showed moderately differentiated (intestinal-type based on Lauren’s classification) adenocarcinoma invading the subserosa (T3). There was evidence of metastasis to regional lymph nodes (N2), and the tumour was classified as pT3pN2(3/19) M0, stage IIIA. Further treatment was recommended, but the patient declined adjuvant chemotherapy. Regular follow-up based on imaging, gastroscopy, colonoscopy, and laboratory examinations indicated no specific findings. At the 14-month postoperative follow-up visit, gastrofibroscopy showed a tumour (size: 2.3*2.5 cm) in the blind jejunal loop of Roux-en-Y anastomosis (Fig. 1C), which was confirmed as adenocarcinoma through an endoscopic biopsy. The PET-CT scan only showed blind jejunal loop uptake (max. SUV, blind loop-9.4) without evidence of any other metastasis (Fig. 2C). The patient underwent segmental resection of the blind loop jejunal cancer, with no gross metastatic lesions within the peritoneum. Pathologic examination showed moderately differentiated adenocarcinoma with angiolymphatic invasion. We sought to determine why, in this case, the cancer was in the jejunal loop. First, we suspected recurrence after gastric cancer surgery. We decided to review all the test results. They all indicated relatively moderately differentiated adenocarcinoma of intestinal type (Fig. 3 Gastric cancer, H&E, ×100). Immunohistochemical (IHC) staining showed homogenous high levels (3+) of CDX2 expression (Fig. 3A–C) in the primary colon cancer and gastric and jejunal adenocarcinomas. Further IHC staining for cytokeratin (CK) profiles demonstrated the same immunoreactivity in both the primary colon cancer as well as the gastric and jejunal adenocarcinomas: CK20(+) and CK7(−) (Fig. 3A–C). The above-mentioned findings strongly suggest sequentially developed metastatic gastric and jejunal cancer with basically the same morphological features and IHC characteristics as those of the primary colonic tumour.

## 3 Discussion

Colorectal cancer is one of the most common cancers in the world. Although various surgical methods and targeted anticancer therapies have led to a decline in mortality, the mortality rate remains high due to metastasis. The most common metastatic sites of colorectal cancer are the liver, lung, and peritoneum. Colon cancer rarely spreads to other sites, such as the bones, spleen, and brain [3]. Gastrointestinal metastasis from colorectal adenocarcinoma has rarely been reported. Disibio et al. [4] reported a metastasis rate of 0.8% to the stomach in a large-scale autopsy study of the metastatic pattern of cancer in 2008. Metastatic carcinomas involving the gastrointestinal tract are mainly melanoma, lung cancer, oesophageal cancer, and breast cancer [5,6].

The endoscopic findings of metastatic tumours to the stomach also vary. Kim et al. reported that metastatic carcinoma has a submucosal tumour-like appearance (32.5%) and a primary carcinoma-like lesion (mucosal origin) (67.5%) [7]. A report by Oda et al. revealed that submucosal tumour-like metastasis (51%) was more common than primary carcinoma-like lesions (39%) [5]. Therefore, it is difficult to distinguish primary gastric cancer from metastatic cancer with a gastroduodenoscopy. Fortunately, there is no major problem in the diagnosis of gastric cancer, among which different types of carcinoma other than adenocarcinoma are distinctly diagnosed. However, when diagnosed as adenocarcinoma or undifferentiated carcinoma, especially in primary gastric cancer cases in Korea, the clinician first considers the primary rather than the metastatic gastric cancer.

The diagnosis of metastatic adenocarcinoma should be carefully considered for the possibility of metastatic lesions. In addition, IHC is required for accurate differential diagnosis. In this study, we found that the adenocarcinomas were derived from colon cancer using CK7, CK20, and CDX2 staining during the diagnosis of metastatic colon cancer. The IHC markers used for the differential diagnosis of metastatic colorectal cancer are usually CK7, CK20, CDX2, and thyroid transcription factor 1 (TTF-1) [8]. Most metastatic carcinomas are treated with chemoradiation therapy. Treatment should be palliative particularly if there are symptoms such as bleeding or obstruction. Nevertheless, the prognosis is poor.

Although metastasis to the small bowel is uncommon, lung and breast cancers are known to primarily metastasize to this location. In particular, small bowel metastasis of colorectal cancer has been reported in some cases. The major route of metastasis to the small bowel is peritoneal carcinomatosis. However, as our report clarifies, intraluminal metastasis due to haematogenous dissemination is not common. In a series by Yutaka et al., seven patients with haematogenous small bowel metastasis displayed no difference in sex or site (jejunum, ileum) [9]. The lesions were mostly moderately differentiated adenocarcinoma invading the subserosal wall. Symptoms of metastasis to the small bowel are mostly obstruction and bleeding. Because treatment is suggested according to symptoms rather than by specific examination, metastatic carcinoma of the small bowel is diagnosed late, and the prognosis is poor.

We reported a case of sequential metastasis to the stomach and jejunum without metastasis to other organs. Eventually, the patient died from brain metastasis.

## 4 Conclusion

Although the exact mechanism of intraluminal metastasis of gastrointestinal tract cancer is not known, IHC staining might prove useful in sequentially metastatic cases when a differential diagnosis must be assessed on consecutive biopsies. To the best of our knowledge, this is the first case of intraluminal metastasis due to colon cancer spreading to the stomach and jejunum. When uncommon carcinoma develops, researchers will need to consider previous carcinoma, as described in this case report.

## Declaration of Competing Interest

The authors declare that we have no conflict of interest.

## Funding

This study did not receive any funding support.

## Ethical approval

This is a case report; therefore it did not require ethical approval from an ethics committee.

## Consent

Informed written consent was obtained from the patient for publication of this report and any accompanying images.

## Author contribution

Lee WY and Lee HK were attending doctors for the patient. Lee WY performed the surgical operation and Lee HK prepared the pathologic report. Lee WY and Lee HK organized the report and wrote the paper. All authors were involved in drafting and revising the manuscript, and all authors read and approved the final manuscript.

## Registration of research studies

Not applicable.

## Guarantor

Woo Yong Lee.

## Provenance and peer review

Not commissioned, externally peer-reviewed.
